# Quantification of Total HIV DNA as a Marker to Measure Viral Reservoir: Methods and Potential Implications for Clinical Practice

**DOI:** 10.3390/diagnostics12010039

**Published:** 2021-12-24

**Authors:** Simone Belmonti, Simona Di Giambenedetto, Francesca Lombardi

**Affiliations:** 1 Dipartimento di Sicurezza e Bioetica Sezione Malattie Infettive, Università Cattolica del Sacro Cuore, 00168 Rome, Italy; simone.belmonti@policlinicogemelli.it (S.B.); simona.digiambenedetto@policlinicogemelli.it (S.D.G.); 2 UOC Malattie Infettive, Fondazione Policlinico Universitario A. Gemelli IRCCS, 00168 Rome, Italy

**Keywords:** HIV, HIV DNA, total HIV-1 DNA, reservoirs, real-time PCR, digital droplet PCR

## Abstract

The focus of this review is to examine the importance of quantifying total HIV DNA to target the HIV reservoir and the clinical implications and challenges involved in its future application in clinical practice. Despite intrinsic limitations, the quantification of total HIV DNA is currently the most widely used marker for exploring the HIV reservoir. As it allows estimating all forms of HIV DNA in the infected cells, total HIV DNA load is the biomarker of the HIV reservoir that provides most of the insights into HIV pathogenesis. The clinical role of total HIV-DNA in both untreated and treated patients is extensively supported by important lines of evidence. Thus, predictive models that include total HIV DNA load together with other variables could constitute a prognostic tool for use in clinical practice. To date, however, this marker has been primarily used in experimental evaluations. The main challenge is technical. Although the implementation of droplet digital PCR could improve analytical performance over real-time PCR, the lack of standardization has made cross-comparisons of the data difficult. An effort by investigators to compare protocols is needed. Furthermore, the main effort now should be to involve the biomedical industry in the development of certified assays for in vitro diagnostics use.

## 1. Introduction

In most patients infected with HIV, the use of combined antiretroviral therapy (ART) results in sustained control of viral replication [1]. However, the virus persists in some specific, latently infected cell-lineages that carry HIV-integrated DNA in their host genome, thus forming the viral reservoir [2,3].

If ART is interrupted, the virus reemerges from the reservoir, leading to a rebound in viremia [4].

Therefore, at the moment HIV cannot be eradicated from the body, and treatment must be maintained indefinitely. Since the reservoir constitutes the major obstacle to eradication of the HIV infection, an accurate and precise measurement of the HIV reservoir is needed to assess strategies for a cure, i.e., a functional or a sterilizing cure [5], and for pilot eradication studies [6]. Quantifying the HIV reservoir is crucial to assess the efficacy of treatments aimed at reducing it and monitoring its changes following specific treatment strategies in studies of intensification, interruption or switching of ART [7]. In particular, measuring the HIV reservoir could be crucial for evaluating patients who are candidates for a simplification of ART, because reducing the drug pressure might be safer in patients who have a small sample-size reservoir. This is noteworthy given the growing importance of less-drug regimens (such as dual regimens) in the management of HIV infection [8].

Thus, a reliable and practical marker is needed to analyze the reservoir persistence and dynamics in HIV-infected subjects. Several markers have been proposed, but there is no consensus regarding the marker that can best measure the reservoir [9,10,11]. Indeed, there are several main questions and difficulties that need to be addressed to define and measure the HIV reservoir. Primarily, according to one definition that has been proposed, the HIV reservoir can be considered “the cells where HIV persists in latent form but can reactivate in the form of a replication-competent virus” [12]. However, it should be considered that only some of these latently infected cells contain replication-competent HIV DNA and can be induced ex vivo to produce the virus; in fact, most of these cells harbor defective viral genome [11,13,14,15,16]. Memory CD4+ T cells are those most infected, even though different T-cell subsets have been found to harbor latent infection and residual virus has also been detected in different cells during ART [17,18].

Furthermore, the different species of HIV DNA include integrated HIV DNA and unintegrated forms such as the linear pre-integration complex and the 1-long terminal repeat (LTR) and 2-LTR circles [19,20].

Moreover, the latently infected cells are disseminated throughout the body but are more concentrated in sites such as the so-called “anatomical reservoirs” (or “sanctuaries”) than in the blood, which is the most accessible organ for sampling [21,22].

Considering these main questions and difficulties concerning the different markers of the HIV reservoir, to date total HIV DNA is the marker that is most widely used and studied. Total HIV DNA is considered an imperfect marker of the HIV reservoir as it is a global biomarker that includes integrated and non-integrated viral genomes, without differentiating the defective forms from the latent ones that can produce infectious viruses. In any case, the clinical role of total HIV DNA has been evaluated in important studies and is supported by the most evidence.

The focus of this review is total HIV-1 DNA—here we examine the importance of quantifying total HIV-1 DNA to target the HIV reservoir, and the clinical implications and challenges involved in its future application in clinical practice.

## 2. Why Is Total HIV DNA the Most Widely Used Marker of the HIV-Reservoir?

The assay that has been considered to represent the gold standard for measuring the latent, but replication competent, virus is the quantitative viral outgrowth assay (QVOA); it quantifies the number of resting CD4+ T cells releasing infectious virus after in vitro stimulation and subsequent co-culture with feeder cells [23,24,25,26].

The great value of this assay is that it can detect individual latently infected cells that release the replication-competent virus, thus providing a valuable estimation of blood reservoir productive activity. However, the major limitations of using this technique are that it is expensive and very labor intensive, even with shortened protocol [23,24,25].

It also involves large amounts of blood (120–180 mL), often requiring leukapheresis. Moreover, it cannot be performed with tissue biopsies and has been demonstrated to have a wide coefficient of variation, indicating that it is unreliable for detecting small differences in the size of the HIV reservoir [27]. Finally, this technique tends to measure only the “tip of the iceberg”, thus underestimating the reservoir, because not all replication-competent latently infected cells can be induced by stimulation [15].

Given these limitations, more reliable, less complex methods are certainly needed for serial measurement of the HIV reservoir in patient management and in clinical studies.

Intracellular HIV RNA load, or cell-associated HIV RNA, is another marker which has been proposed as a marker of HIV transcription in latently infected cells [28]. Indeed, its expression has been used to demonstrate that several drugs could induce latently infected cells from patients under suppressive ART to make HIV transcripts [29]. Therefore, quantification of multiple spliced, singly spliced and unspliced HIV RNA has also been proposed as a marker of the “active HIV reservoir”, i.e., of a subset of the total viral reservoir containing cells in which HIV sequences are actively transcribed, and as a marker of residual virus replication [28].

However, whether some transcription is consistent with the latent status or represents ongoing low-level replication must still be determined. Several assays have also been developed to quantify intracellular HIV RNA [28], but their reliability and reproducibility must still be fully evaluated.

The quantification of total HIV DNA permits estimating all forms of HIV DNA in the infected cells (i.e., resting or activated) that are present in blood as well as in biopsies and tissues. One of the major criticisms regarding the quantification of total HIV DNA is that it does not distinguish between latent defective and replication competent forms.

It has been hypothesized that defective proviruses are probably generated during the initial rounds of replication after transmission, because defective proviruses are generated at a high frequency from the process of reverse transcription. Similarly, it has been hypothesized that defects such as deletions probably occur during minus strand synthesis before the second strand transfer event of reverse transcription [11,15,16]. The most common defects are internal deletions, which varied in size and in location in the genome. The very large deletions (>6Kb) can encompass most of the HIV genome. Some proviruses show 3′ deletions affecting the *env*, *tat*, *rev* and *nef* genes, other proviruses show 5′ deletions affecting *gag* and *pol* genes. A small percentage of proviruses can have small deletions at the packaging signal and the major splice donor site represented. These latter proviruses are probably replication defective owing to a failure to correctly make spliced HIV-RNAs or to package genomes into virions [15,16]. Some clones can contain multiple deletions. A percentage of proviruses harbor guanine-to-adenine (G-to-A) hypermutations. An additional percentage of proviruses can contain both deletion and hypermutation indicating that these processes can occur contextually during the reverse transcription. Hypermutation in turn can alter start codons and/or introduce stop codons in the larger open-reading frames (ORFs) (*gag, gag–pol, env* and *nef*). All these defects and deletions affecting key viral ORFs probably prevent many defective proviruses from being eliminated either by viral cytopathic effects or by eradication strategies that depend on viral protein expression [15,16].

However, if quantifying all forms of HIV-DNA in the infected cells can be considered a structural limit on one hand, on the other it can be considered an advantage. In fact, each of the various forms that are quantified in total HIV DNA play a different role in HIV pathogenesis [30]. This participation can occur through the creation of new cycles of infection and infected cells, with the maintenance /amplification of the reservoir by homeostatic proliferation and through viral transcription, thus producing viral antigens and incomplete viruses without any new production of virions. These proteins can thus induce immune activation/inflammation, thereby participating in maintaining the cycle of HIV pathogenesis and in facilitating the persistence of HIV reservoirs [30].

From this point of view, the key question is how to consider the reservoir. If the HIV reservoir can be defined as all infected cells and tissues containing all forms of HIV persistence, the quantification of total HIV DNA is the biomarker of the HIV reservoir that provides most of the insights into HIV pathogenesis.

On the other hand, several studies have shown that the total HIV DNA load seems to correlate well with the frequency of cells containing the replication-competent virus. Studies by Eriksson et al. [11] and Kiselinova et al. [31] pointed out a correlation between total HIV DNA load in resting cells and quantification of the replication-competent virus. In a different study, Noel et al. reported that a low level of total HIV DNA correlated with low efficiency of virus production after activation [32]. The second main criticism is that the quantification of total HIV DNA does not discriminate between integrated and unintegrated forms. Although the integrated DNA (provirus) is transcribed and leads to synthesis of viral proteins and completion of the viral replication cycle [33], the unintegrated forms cannot lead to a productive infection by themselves [34]. These unintegrated species include linear DNA, which is a component of a pre-integration complex [33], and circular forms, which derive from the previous by means of several mechanisms. The two main circular forms are 1-LTR and 2-LTR, which harbor one and two LTR extremities of cDNA, respectively [34]. Although the proportion between integrated and unintegrated forms can vary in vitro [35], in vivo during ART it appears to be very similar, given the clearance of non-integrated HIV DNA when ART is initiated [35,36,37]. On the other hand, an accumulation of unintegrated forms has been observed in ART-naïve subjects [38]. Although the unintegrated forms cannot be copied during cell division, transcription activity has also been shown from linear DNA and 1-LTR and 2-LTR circles [34,39].

Circular forms of DNA are more stable than linear forms, even though unintegrated forms are generally much more labile compared to integrated DNA and have a short half-life [35]. Thus, the quantification of integrated DNA, which is the most stable form of HIV-DNA and serves as the main template for gene transcription and viral replication and is important for viral latency is thought to be the best indicator of the HIV reservoir size. However, it presents several main concerns. The first is technical. Indeed, assays specifically targeting integrated HIV DNA are complex to set up and require extensive replicate testing [11,40]. Furthermore, it has been demonstrated that extrachromosomal forms can be associated with transcription and can contribute to HIV pathogenesis [34,39,41], thus making total HIV DNA a better indicator of HIV persistence. Moreover, due to the clearance of unintegrated forms during ART [35,36,37], it should be considered that total and integrated HIV DNA levels became generally equal in effective ART [37].

Therefore, even though the quantification of integrated and unintegrated forms can provide information in select studies, it does not appear reliable for routine use as an HIV reservoir marker. Instead, the quantification of unintegrated forms has been proposed as indicative of ongoing viral replication [42,43]; in any case, controversies remain.

Through comparison with these markers, total HIV DNA has the advantage of easier quantification by the quantitative real-time (qPCR) or digital droplet PCR (ddPCR) assays. The measure of total HIV DNA load by the PCR-based assays is quick, easy to perform and has a large dynamic range of quantification. Compared to other assays, the quantification of total HIV DNA can be carried out in blood, other body fluids and in tissue biopsy specimens. It requires a small amount of the sample and is unaffected by freeze-thawing; it also seems to be more cost-effective, less time consuming and easy to implement in the laboratory [44,45,46,47,48,49,50,51,52,53,54,55].

Therefore, total HIV DNA cannot be considered a perfect maker as it is neither a marker of the replication-competent reservoir, such as the QVOA, a marker of the latent reservoir, such as the integrated HIV DNA, nor a marker of the active reservoir, as is the cell-associated HIV RNA. However, for the above-mentioned reasons, the quantification of total HIV DNA is currently the most widely used marker for exploring the HIV reservoir.

Novel approaches such as IPDA [56] Q4PCR [57] and TILDA [58] represent major advances in quantifying and characterizing intact/replication-competent HIV reservoir. The implementation of these assays might constitute innovative methods for the analysis of the viral reservoir in the future.

Emerging PCR-based techniques used to quantify HIV DNA such as the intact proviral DNA assay (IPDA) and quadruplex quantitative PCR (Q4PCR), distinguish intact proviruses from defective ones, therefore providing a better resolution for studying the dynamics of defective and intact HIV-proviral DNA and requiring a relatively small number of cells.

IPDA is a high-throughput assay that uses two probes to exclude the majority of defective proviruses and to determine the frequency of intact proviruses, albeit without sequence confirmation.

The IPDA utilizes ddPCR to measure proviruses with probes targeting conserved regions. This duplexed ddPCR assay simultaneously targets two HIV-1 regions, the Packaging Signal (Ψ) near the 5′ end of the viral genome and the Rev Responsive Element (RRE) within Envelope (*env*), which when amplified together in the same provirus, includes only genomically intact provirus and excludes the majority of defective proviruses. Input cells are directly measured by a simultaneous ddPCR reaction, enabling the IPDA to report the total frequency of intact and defective proviruses per million input cells. This assay enables much more specific quantification of intact proviral DNA than single-probe assays. However, the Ψ and *env* probe combination may not be entirely predictive of a fully intact provirus [56,59].

Recent studies have described instances of assay failure, attributable to natural HIV polymorphism in primer and/or probe binding regions. [60].

The Q4PCR assay employs long distance PCR at limiting dilution to amplify proviruses, followed by interrogation with four qPCR probes for Ψ, *env*, *pol*, and *gag* in a multiplex reaction to detect amplified HIV genomes. Cell inputs are estimated by quantification of input DNA, and the frequency of intact proviruses is reported after sequence verification through near-full-length genome sequencing (nFGS). Because the Q4PCR employs limiting dilution near-full-length proviral amplification, these nFGS results can be used to provide insight into the clonal composition of intact proviruses [57]. However, inefficiencies in long-distance PCR amplification of proviruses in Q4PCR may lead to underestimates of intact proviral frequencies [57,59].

Notably, proviral intactness does not guarantee virion production and the fraction of intact proviruses that can be induced to produce virions cannot be determined by these assays, IPDA and Q4PCR, which is a critical limitation given that many intact proviruses exhibit low inducibility [15,61].

The transcription-competent (inducible) HIV reservoir can be quantified by several methods including a well-established inducible reservoir assay, *tat/rev* induced limiting dilution assay (TILDA). Data indicate that there are circuits enabling control of viral transcription without cellular activation and showing specifically that Tat positive feedback is sufficient to regulate latency independent of cellular activation [62]. TILDA measures the frequency of cells that harbor viral genomes, and which produce tat/rev multiply-spliced HIV RNA upon maximal stimulation, reducing the likelihood of quantifying defective genome. This method allows to measure the frequency of cells capable of being induced to produce HIV RNA transcripts by combining ultra-sensitive detection of msRNA and maximal activation of CD4+ T cells in a limiting dilution format [58].

However, due to post-transcription blocks in RNA processing not all cells producing tat/rev msRNA transcripts will yield infectious virus; therefore, quantifying these cells can result in overestimating the replication-competent HIV reservoir size [63,64].

TILDA holds great promise for application in clinical trials because it requires less than 1 million viable target cells per condition and can be completed within two days.

**Figure 1:**
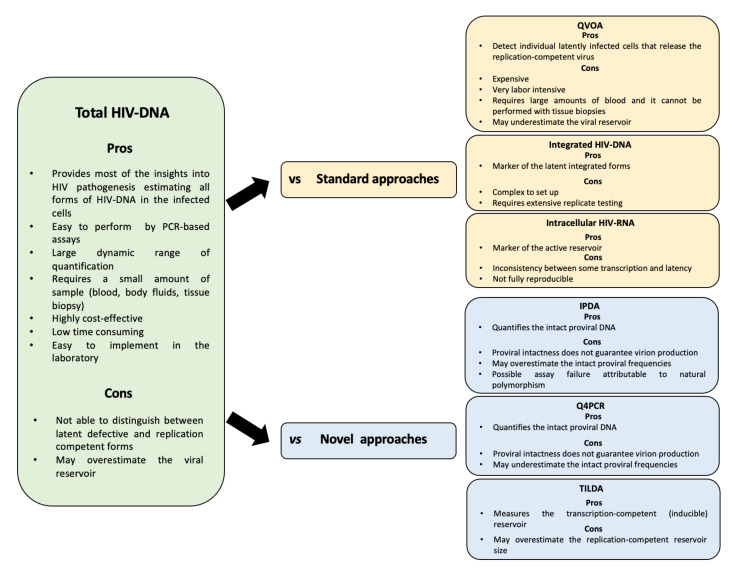
Total HIV DNA vs other approaches for quantifying the HIV-reservoir.

Figure 1 summarizes and compares pros/cons of total HIV DNA quantification *vs* pros/cons of other approaches for quantifying the HIV reservoir.

## 3. Technical aspects

Technical concerns regarding measurement of total HIV DNA include the specimens and the assays.

### 3.1. The Specimens

One of the major criticisms concerning measurement of the HIV reservoir is the distribution of latently infected cells in the different organs and tissues. Blood is the most accessible organ as it can easily be obtained by venipuncture; other tissues are not as easily sampled. Therefore, most studies have explored blood-associated total HIV DNA despite concerns as to whether it is representative of the latent reservoir size in the whole body. In fact, it has been shown that most infected cells can be found in the lymph nodes and the gastrointestinal tract [65,66,67,68], and the activation status of the latently infected CD4+ is higher in these sites than in the blood [67]. However, a phylogenetic analysis carried out by Josefsson L. et al. revealed genetic similarity of HIV DNA in CD4+ T-cells from lymph nodes and peripheral blood, which indicates an interchange of infected cells among these compartments during infection [69]. Furthermore, in a different study it was noted that total HIV DNA load in rectal cells correlated with the blood-associated total HIV DNA levels [70]. However, this finding was not confirmed in other reports [66,67]. On the other hand, some authors considered how the blood HIV DNA could be generally accepted as a reflection of the entire HIV reservoir in the same way that blood HIV RNA or the CD4+ T cell count are generally accepted as a reflection of virus activity in the whole body [71].

Quantification of the total HIV-DNA levels in the blood can be performed directly on whole blood cells or can require a more laborious step by separation of PBMCs or by purification of CD4+ T cells. Depending on the method used the results are expressed as copies/leukocytes, copies/PBMCs or copies/ CD4+ T cells, respectively. Some studies found a good correlation between the results of the whole blood sample tests for total HIV DNA and those of PBMC samples [49,72]. Another study found that HIV DNA load could predict disease progression regardless of the mode of results expression [73].

### 3.2. The Assays

Historically, the method most used to measure total HIV DNA is qPCR. In this assay, amplification is carried out with primers and probe targeting of a conserved region of the HIV genome; quantification is assessed to calculate the frequency of infected cells based on a standard curve prepared by serial dilution of a standard (i.e., 8E5 cell line) [44,45,46,47,48,49,50,51,52,53].

However, there are no certified methods for in vitro diagnostic use and several in-house protocols have been developed. These homebrew HIV DNA quantification protocols may differ in the different genomic regions analyzed and in the different standards, probes and control genes used.

Total HIV-DNA quantification protocols have been developed using both Taqman probes and SYBR green-based PCR, targeting LTR region, gag-pol junction, gag gene or pol gene [44,45,46,47,48,49,50,51,52,53]. An alternative method uses two sets of primers that target the genomic regions of LTR and gag by molecular beacon probes [74].

The methods that target the conserved region of the long terminal repeat have been reported to have more sensitivity, specificity, and reproducibility than the others [75,76]. Other protocols that have been proposed to quantify total HIV DNA using technologies such as Nested- PCR or semi-nested PCR [53,77] are used less frequently. Thus, in the last two decades the qPCR has become the tool most used for the quantification of total HIV-DNA. Despite its success, qPCR remains an indirect measurement tool. It requires a validated standard curve and is susceptible to small changes in the efficiency of the reaction, exponentially amplifying the noise and making it difficult to differentiate target low levels from assay noise. dPCR is a more recent alternative to real-time PCR for the measurement of total HIV DNA load. This technology allows for absolute quantification of the target nucleic acid without reliance on the use of an external standard. Indeed, in this technique each sample is divided into thousands of independent microscopic “end-point” PCR reactions so that only one or no nucleic acid molecule will be found in each partition [78]. In such a this sufficiently diluted sample, the template follows the POISSON distribution, which can be used to derive the target concentration based on the frequency of the positive and negative distributions. The high costs and labor-intensive procedure underlying the micro partitioning have historically limited the use of dPCR. Developments in the field of microfluidics have made digital PCR platforms commercially available in a low-cost and practical format.

Micro partitioning can be achieved by emulsification of the aqueous PCR reaction mixture in a thermostable oil, as in droplet digital PCR (ddPCR) [79]. This technique was applied to total HIV DNA quantification and showed high accuracy and precision [54,55].

When qPCR and ddPCR techniques were compared in a study conducted by Heinrich et al. [80], they showed a similar level of analytical sensitivity. In a different study, ddPCR provided a significant increase in precision compared to qPCR; moreover, additional benefits of the ddPCR over the qPCR assay included relative insensitivity to mismatches in primer and probe sequences [54]. Accordingly, Bosman et al. compared ddPCR platforms with a semi-nested qPCR and showed a higher reproducibility for ddPCR than qPCR; however, some false-positive signals were observed with ddPCR [81]. The sensitivity of the ddPCR assay makes it advantageous for analyzing molecules with a rare frequency, i.e., in the presence of a high background of non-target molecules. Theoretically, this makes it an ideal model for studying the HIV reservoir.

## 4. The Clinical Relevance of Total HIV-DNA Load

Multiple studies have demonstrated the clinical relevance of quantifying total HIV- DNA load. This marker has been used extensively in studies of HIV reservoir dynamics in both treated and untreated subjects.

### 4.1. During Infection

Results of several relevant studies support the predictive value of the measure of total HIV DNA level during the natural history of HIV infection. In fact, total HIV DNA load has been shown to be predictive of disease progression independently of HIV RNA load and the CD4+ T cell count in untreated HIV-positive subjects [82,83,84,85,86,87].

Rouzioux et al. showed that total HIV DNA level was a major predictor of progression to AIDS independently of HIV RNA level and CD4+ T cell count from 6 to 24 months after seroconversion [82].

A meta-analysis of six studies of untreated patients reported that total HIV-DNA was a better predictor of progression to AIDS and of all-cause mortality than plasma HIV-RNA load in untreated patients [86].

In another more recent study, N’takpe et al. analyzed the association between blood-associated total HIV DNA load and long-term mortality in African adults who started ART early. The authors found that patients with a high HIV DNA level at baseline had a higher 6-year mortality risk than other patients, irrespective of plasma viral load, CD4+ T cell count and ART [87].

A different study showed that total HIV DNA in blood can be detected very early after infection, with high levels seeded in primary infection at the peak and with the HIV- DNA set point rapidly established [88,89]. Furthermore, total HIV DNA load has been shown to be significantly lower in patients with primary infection, i.e., at stage Fiebig I versus Fiebig II–IV [90]. In the ANRS PRIMO cohort, the median HIV DNA load was significantly higher in patients who had a symptomatic primary infection [91].

Elite controllers and long-term non progressors are characterized by a very low and stable cell-associated HIV-DNA level [32,92,93]. Total HIV DNA levels in the blood correlated positively with plasma viremia and negatively with CD4+ T cell count [82]. Moreover, a link has been observed between HIV DNA levels and T-cell activation [94] and in primary infection early levels of HIV DNA predict the extent of T-cell proliferation [95]. Furthermore, the HIV DNA load plays an important role in the pathogenesis of HIV-associated neurological disorders. In fact, some studies reported that the blood-associated total HIV DNA level is associated with the occurrence and severity of HIV-associated neurological disorders [96,97].

Finally, of note, the quantification of total HIV DNA load in the peripheral reservoir of monocytes was shown to be predictive of cognitive performance in an elegant study by Valcour VG et al. [98].

### 4.2. During ART

There is evidence that the earlier ART begins, more prominent is the decrease in total HIV DNA load [88,89]. Furthermore, unlike the slow decrease that is observed in patients treated at the chronic stage, this decay is faster in acutely infected patients receiving ART. Indeed, several studies showed that total HIV DNA levels dropped rapidly during the first year of ART and then showed a subsequent slower decline from years 1 to 4 until they reached a plateau phase where no further decay was observed [99,100]. However, several more recent studies reported that after several years of suppressive ART, HIV DNA levels could continue to slowly decline [101,102,103]. The authors suggest that this decay of HIV DNA during effective ART could reflect the ongoing loss of HIV-infected cells partially counterbalanced by clonal proliferation of infected cells [101,102]. In any case, this point needs further investigation.

It has been shown that total HIV- DNA load during ART reflects pre-ART characteristics of infection, such as baseline HIV- DNA load, pre-ART HIV-RNA load and the CD4+ cell count nadir [104,105,106]. Furthermore, total blood-associated HIV- DNA levels correlated with the levels of residual plasma viremia in patients under suppressive ART [107,108].

It has also been reported that the presence of X4-tropic viral DNA variants is related to a larger size of the HIV DNA blood reservoir compared to the presence of R5-tropic viruses in patients fully responding to ART [109]. In a different setting, total HIV-DNA load was not associated with soluble biomarkers of inflammation in treated patients [110].

Lower baseline HIV-DNA levels were predictive of achieving a sustained virological response on ART [108,111], whereas several reports showed that baseline HIV DNA load predicted the level of residual plasma viremia during effective ART [112,113].

In a recent study baseline total HIV-DNA and CD4+ T cell count resulted to be independent predictors of total HIV-DNA after treatment [114].

Of note, it has been demonstrated that the measurement of total HIV DNA load is informative when measured at the time of treatment interruption, as it is the only biomarker predictive of the time to plasma HIV RNA rebound after treatment interruption, both in patients treated early during primary infection and in patients treated late during chronic infection; low levels predict a higher probability of maintaining viral control [84,115,116,117].

In particular, the study by Williams JP. et al. measured total HIV DNA load in a highly characterized randomized population of individuals with primary HIV infection. Results showed that those with higher levels of HIV DNA when the treatment was stopped experienced faster viral rebound than those with lower levels of HIV DNA [84].

Instead, the study by Assoumou L. et al. evaluated whether HIV replication could be controlled following interruption of treatment started early in the course of infection, but not during the primary infection. In this study, patients who had low HIV DNA levels at the time of treatment interruption maintained viral control for long periods [116]. In addition, the study by Piketti C. et al. predicted a shorter time to treatment resumption, independently of the CD4 nadir in patients treated during chronic infection [117].

A specific topic deserves discussion here, i.e., the strategies of de-escalation therapies and the role of HIV DNA load in this setting. The use of less-drug regimens seems to indicate a new era of significant changes in the management of ART for people living with HIV. These therapies aim to combine virological efficacy with the limited long-term toxicity associated with use of the nucleos(t)ide reverse transcriptase inhibitors (NRTIs), limited adverse effects and reduced costs when compared to standard three-drug ART [8,118]. Within this context, the quantification of total HIV DNA levels could prove helpful for choosing the best candidates, in whom it may be safer to reduce drug pressure. Furthermore, it could be useful for evaluating these new strategies in terms of effective action on the reservoir.

Of note, several studies have demonstrated the predictive value of total HIV DNA load on the risk of virological failure following treatment de-escalation. Indeed, the total HIV DNA levels at the time of a switch to ritonavir-boosted darunavir (DRV/r) monotherapy predicted the risk of viral rebound at week 96 in the MONOI trial [119].

Accordingly, in the MONET randomized study Geretti et al. showed that baseline HIV DNA levels were higher in patients who switched to DRV/r monotherapy and who had at least a viral rebound during the 144 weeks of follow-up [120].

In addition, higher blood-associated HIV DNA copy number at the time of a dolutegravir (DTG)-based monotherapy initiation, was associated with virological failure in the DOMONO trial [121].

In the setting of virological suppression, some two-drug regimens (2DRs) have demonstrated comparable effectiveness to standard triple ART for maintenance therapy. Thus, several studies evaluated the impact of 2DRs as maintenance/switch treatment on the cellular reservoir size [8,118]. A sub study of the ATLAS-m trial compared the impact of 48 weeks of atazanavir/ritonavir plus lamivudine dual therapy versus maintaining the atazanavir/ritonavir plus two NRTIs regimen on total HIV DNA levels and found a similar decline in the two study groups [122].

When compared to other virologically effective 2DRs, the combination of DTG plus lamivudine (3TC) had several advantages in terms of lower risk of drug interactions and metabolic complications. Furthermore, it has shown excellent viro-immunological properties in both naïve and ART-experienced HIV-infected patients [8,9,10,11,12,13,14,15,16,17,18,19,20,21,22,23,24,25,26,27,28,29,30,31,32,33,34,35,36,37,38,39,40,41,42,43,44,45,46,47,48,49,50,51,52,53,54,55,56,57,58,59,60,61,62,63,64,65,66,67,68,69,70,71,72,73,74,75,76,77,78,79,80,81,82,83,84,85,86,87,88,89,90,91,92,93,94,95,96,97,98,99,100,101,102,103,104,105,106,107,108,109,110,111,112,113,114,115,116,117,118]. Thus, this strategy currently constitutes the most widely prescribed two-drug regimen. An observation from the single arm ANRS167 LAMIDOL trial suggests that, in virally suppressed patients, the treatment switch to DTG plus 3TC from a standard triple regimen could have a favorable effect on the cellular reservoir in terms of control of total HIV DNA levels in the blood after 48 weeks [123]. A different longitudinal, matched, controlled study by Lombardi F. et al. showed that after 48 weeks of treatment this simplification strategy had the same impact on the cellular viral reservoir as the triple regimen [103]. In fact, similar changes in total blood-associated HIV DNA levels were observed in the dual therapy group and the triple therapy group.

The same group conducted a different study on the HIV DNA decay in ART-naïve patients starting treatment with DTG plus 3TC versus a standard DTG-based triple regimen. The preliminary study findings seem to suggest that, at least in the short term, using DTG plus 3TC is not detrimental for controlling the HIV reservoir [124].

**Figure 2:**
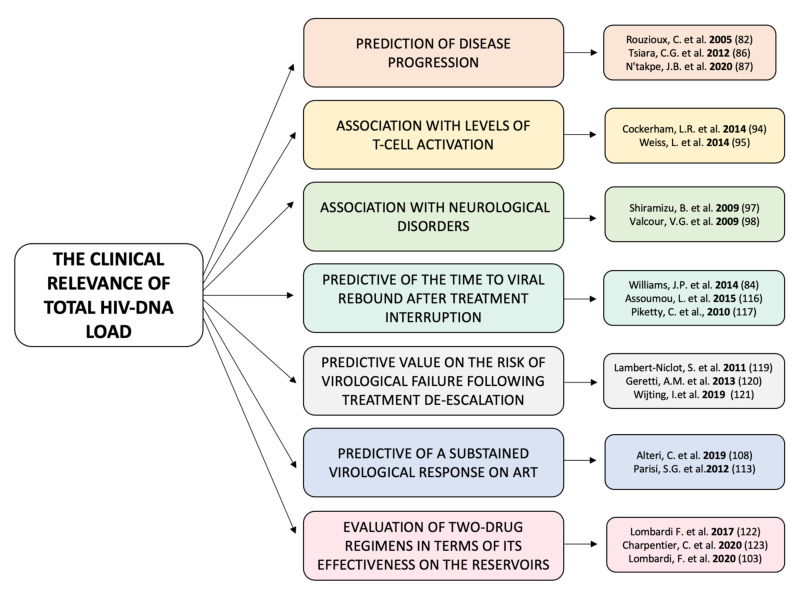
Main studies supporting the clinical role of total HIV-DNA.

Figure 2 summarizes the main studies supporting the clinical role of total HIV DNA.

## 5. What Are the Challenges Now?

Despite intrinsic limitations, the quantification of total HIV DNA load is broadly accepted as a measure of the viral reservoir as well as a promising marker for monitoring its changes following specific treatment strategies.

Since it has been demonstrated that measuring the levels of total HIV-DNA makes it possible to estimate the spread of the infection and reflects the ability of antiretroviral therapy to purge reservoirs, it could be an important factor in establishing disease progression and predicting the outcome of ART. It could also be a useful biomarker for monitoring the efficacy of ART, particularly for such novel treatment as long-acting injectable treatments [125,126], as well as novel strategies aimed at reducing or eliminating HIV reservoirs. Furthermore, measuring total HIV DNA load could help identify candidates for therapeutic strategies such as simplification and structured interruption of therapy.

However, data suggest that HIV DNA should not be the only marker used for these clinical purposes. For example, it has been observed that subjects with low HIV DNA levels are not always able to control the infection after interruption of therapy or after simplification.

Therefore, even though HIV DNA should be considered a marker that provides information about the size of the viral reservoir that is not available with the standard markers of HIV RNA load and CD4+ T cell count, this information must necessarily be integrated with that provided by traditional markers and other parameters that are now available (i.e., ultrasensitive plasmatic viremia), as well as clinical data, to better evaluate the disease and to optimize the management of ART.

Predictive statistical models that include HIV DNA together with the other variables might constitute a useful and truly prognostic tool in clinical practice. For example, in this regard some teams derived a predictive score for virological failure to define an appropriate selection of patients for treatment simplification to a dual ART [127]. The inclusion of an important variable, such as total HIV DNA load, could improve the predictive value of the derived score as well as allow validation for clinical purposes.

Although HIV DNA load might be part of clinical practice in the future, to date it is a marker that is mainly used in experimental evaluations. There are no established clinical cut-offs or referencing intervals with a clinical relevance. The main challenge seems to be technical. In fact, to date no certified assay to measure total HIV DNA load has been developed. Few commercial assays have been developed, but they have not been certified for in vitro diagnostic use.

Several “in-house” PCR methods have been used to quantify total HIV DNA worldwide. The assays typically differ as to the standard used, the genomic regions analyzed and/or technique performed, such as real time PCR or droplet digital PCR.

The implementation of droplet digital PCR could improve the accuracy and precision of the total HIV DNA quantification over real-time PCR. However, the heterogeneity of methods has made it difficult to interpret the data generated by different labs because of different levels of sensitivity, accuracy, and precision. Thus, the lack of standardization has made it difficult to carry out cross-comparisons of the data.

There is a need for investigators to compare protocols to permit the validation and standardization of the different methods. In this regard, an effort was made by some teams through multicenter quality controls evaluating the inter-laboratory reproducibility of total HIV DNA quantification [128,129,130].

In any case, the main effort now should be to involve the biomedical industry. The development of certified systems could be the key for implementing the use of total HIV DNA load in routine diagnostic settings.
